# Holistic Assessment of NIR-Emitting Nd^3+^-Activated Phosphate Glasses: A Structure–Property Relationship Study

**DOI:** 10.1021/acsorginorgau.3c00071

**Published:** 2024-03-05

**Authors:** José A. Jiménez

**Affiliations:** Department of Biochemistry, Chemistry, and Physics, Georgia Southern University, Statesboro, Georgia 30460, United States

**Keywords:** phosphate glasses, lasers, optical properties, structural properties, thermal properties

## Abstract

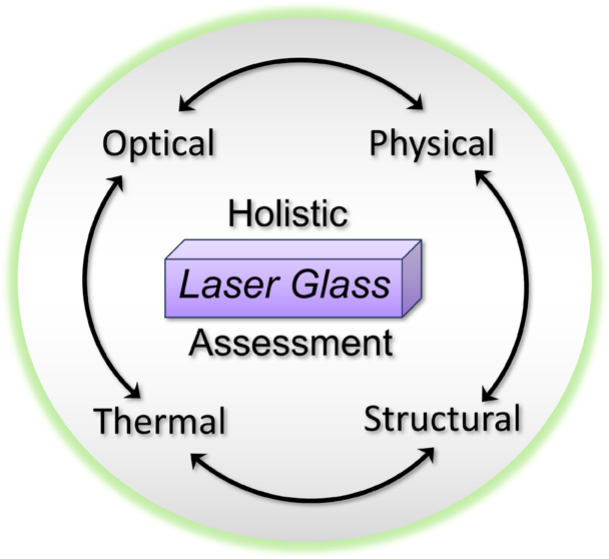

Near-infrared (NIR)-emitting phosphate glasses containing Nd^3+^ ions are attractive for applications in laser materials and solar spectral converters. The composition–structure–property relation in this type of glass system is thus of interest from fundamental and applied perspectives. In this work, Nd^3+^-containing glasses were made by melting with 50P_2_O_5_-(50 – *x*)BaO-*x*Nd_2_O_3_ (*x* = 0, 0.5, 1.0, 2.0, 3.0, 4.0 mol %) nominal compositions and studied comprehensively by density and related physical properties, X-ray diffraction (XRD), Raman spectroscopy, O 1s X-ray photoelectron spectroscopy (XPS), differential scanning calorimetry (DSC), dilatometry, ultraviolet–visible (UV–vis)–NIR optical absorption, and photoluminescence (PL) spectroscopy with decay dynamics assessment. The densities and molar volumes of the Nd^3+^-containing glasses generally increased with Nd_2_O_3_ concentration also resulting in shorter Nd^3+^–Nd^3+^ distances. XRD supported the amorphous nature of the glasses, whereas the Raman spectra evolution was indicative of glass depolymerization being induced by Nd^3+^ ions. Oxygen (1s) and phosphorus (2p) analysis by XPS for the glass with 4.0 mol % Nd_2_O_3_ agreed with the increase in nonbridging oxygens relative to the undoped host. DSC results showed that the glass transition temperatures increased with Nd^3+^ concentration, with the glasses also displaying a decreased tendency toward crystallization. Dilatometry showed trends of increasing softening temperatures and decreasing thermal expansion coefficients with increasing Nd_2_O_3_ content. A glass strengthening/tightening effect was then indicated to be induced by Nd^3+^ with higher field strength compared to Ba^2+^ ions. The UV–vis–NIR absorption by Nd^3+^ ions increased consistently with Nd^3+^ concentration. The UV–vis absorption edges of the Nd-containing glasses were also analyzed via Tauc and Urbach plots for comparison with the undoped host. Concerning the PL behavior, the Nd^3+^ NIR emission intensity was highest for 1.0 mol % Nd_2_O_3_ and decreased thereafter. The decay kinetics of the ^4^F_3/2_ emitting state in Nd^3+^ ions analyzed revealed decreasing lifetimes where the decay rate analysis pointed to the prevalence of ion–ion excitation migration leading to PL quenching at high Nd^3+^ concentrations.

## 1 Introduction

Glasses activated with near-infrared (NIR)-emitting Nd^3+^ (*f*^3^) ions have been the subject of extensive research, given their use in high-power lasers^1−11^ and more recently being interesting in the context of solar spectral converters.^12−14^ Among the numerous glass matrices available, phosphate-based glasses are particularly attractive, given their high metal solubility, suitable thermal properties (e.g., low-melting character), and associated low production costs and manufacturability.^1−10^ Studying the composition–structure–property relationship in this type of glass systems is thus of interest from fundamental and applied perspectives.

Several decades ago, Hayden et al.,^1^ Campbell,^2^ and Campbell and Suratwala^3^ reported extensively on the effect of various alkali/alkaline earth cations in neodymium laser glasses and reported correlations with cations field strength for properties such as thermal expansion, Young’s modulus, refractive index, and emission cross sections. Details pertaining to underlying structural features of the glasses and their connection with properties evaluated were however not the subject of scrutiny.^1−3^ Other recent reports, e.g., Li et al.^5^ and Algradee et al.,^7^ have focused largely on evaluating thermomechanical and physical properties in neodymium-doped phosphate glasses, nonetheless independently from Nd^3+^ luminescent characteristics. Conversely, luminescence-focused works such as the reported by Sontakke et al.^4^ and Ramprasad et al.^9^ have evaluated the optical properties of Nd^3+^-doped phosphate glasses but not assessing other structural and thermal properties of interest to applications. Muñoz-Quiñonero et al.^10^ reported more comprehensively on various optical, structural, and thermal properties of Nd^3+^-doped aluminophosphate glasses. Still, such types of studies encompassing a wide range of characterizations for an in-depth analysis are scarce.^10,11^ Hence, there appear to be opportunities for investigating further the connection of fundamental structural properties assessed, for instance, through spectroscopic techniques together with thermal and optical properties. Of great value in this sense are wide-ranging spectroscopic characterizations which help elucidate the different contributions of the constituents to glass structure and the consequent impact on thermomechanical and optical properties of interest.

The present work was then undertaken with the object of scrutinizing further the structure–property relationship in Nd^3+^-containing phosphate glasses. Barium phosphate was chosen as the glass host matrix since large-radius Ba^2+^ cations are generally considered suitable network modifiers for achieving necessary optical, thermal, and mechanical properties in phosphate glasses for various photonic applications.^3,4,6,14−17^ Hence, using as a starting point, the 50P_2_O_5_-50BaO glass matrix prior considered by the author,^6,14,18^ the glasses were made with 50P_2_O_5_-(50 – *x*)BaO-*x*Nd_2_O_3_ (0 ≤ *x* ≤ 4 mol %) nominal compositions by the melt-quenching technique. A comprehensive experimental investigation followed, encompassing measurements by densitometry, X-ray diffraction (XRD), Raman spectroscopy, X-ray photoelectron spectroscopy (XPS), differential scanning calorimetry (DSC), dilatometry, UV–vis–NIR optical absorption, and photoluminescence (PL) spectroscopy with decay kinetics assessment. The various parameters extracted from measurements were consequently examined in the context of glass structure and differing Nd^3+^ concentrations seeking insights into the origin of the physicochemical behavior.

## 2 Experimental Section

### 2.1 Glass Preparation

**Figure 1:**
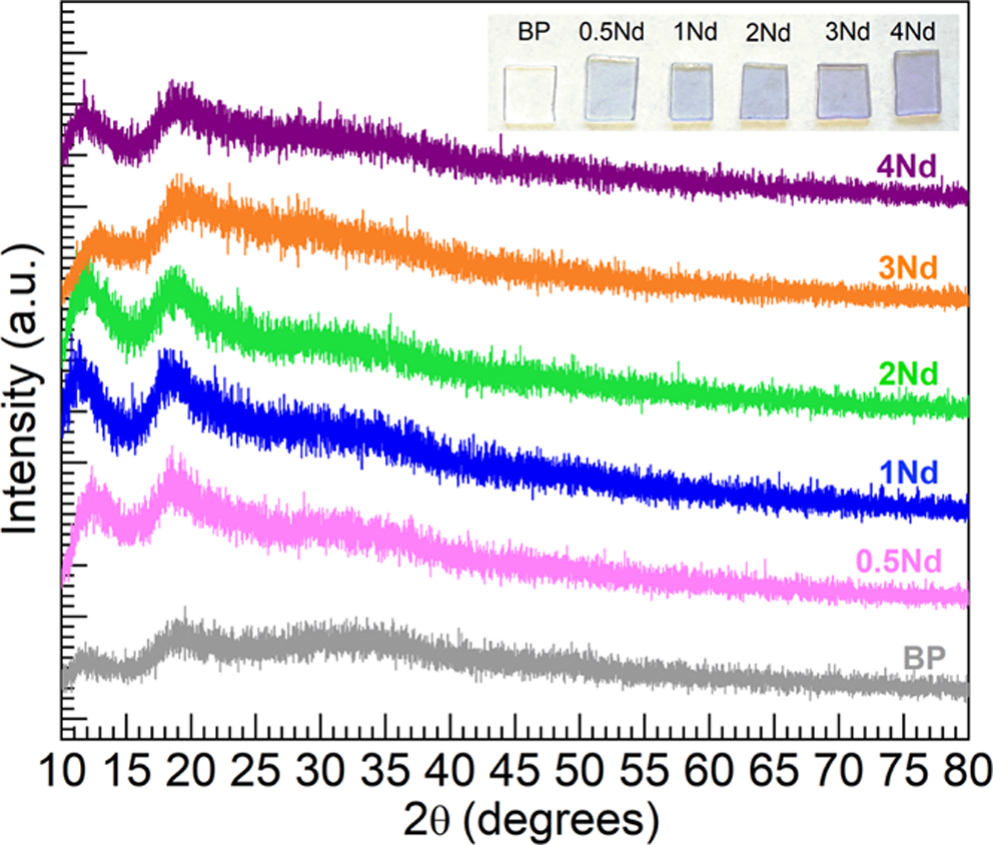
Powder XRD patterns obtained for the various glasses using Mo-Kα radiation. The inset is a photograph of slabs for the different glasses.

The glasses were synthesized by melting with 50P_2_O_5_-(50 – *x*)BaO-*x*Nd_2_O_3_ nominal compositions, where *x* = 0, 0.5, 1.0, 2.0, 3.0, and 4.0 mol %. The raw materials used were high purity reagents P_2_O_5_ (98%), BaCO_3_ (99.8%), and Nd_2_O_3_ (99.99%). The different compounds were weighed in the appropriate quantities (about 25 g batches), thoroughly mixed, and melted under an ambient atmosphere in porcelain crucibles at 1150 °C for 15 min. The melts were swirled after 7 and 15 min and quenched by pouring onto heated steel molds. The glasses were annealed below the glass transition temperature (vide infra) at 420 °C for 3 h to relinquish stress. The glasses were cut and polished to about 1 mm thick slabs for spectroscopic measurements. The glasses had a transparent appearance; the BP glass was colorless whereas the Nd-containing glasses had a purple hue which intensified with increasing Nd_2_O_3_ concentration (a photograph of samples for the different glasses is shown in the inset of Figure 1). Glass samples were also quenched in cylindrical shapes and cut to a length (*L*) of about 2.54 cm for dilatometric measurements. The glass labels and respective nominal molar compositions are summarized in Table 1.

**Table 1:** Glass Codes and Nominal Compositions of the 50P_2_O_5_-(50 – *x*)BaO-*x*Nd_2_O_3_ (*x* = 0, 0.5, 1, 2, 3, 4 mol %) Glasses Synthesized

glass	P_2_O_5_ (mol %)	BaO (mol %)	Nd_2_O_3_ (mol %)
BP	50.0	50.0	
0.5Nd	50.0	49.5	0.5
1Nd	50.0	49.0	1.0
2Nd	50.0	48.0	2.0
3Nd	50.0	47.0	3.0
4Nd	50.0	46.0	4.0

### 2.2 Measurements

#### 2.2.1 Density

Densities were measured for the various glasses by the Archimedes principle with a Mettler-Toledo XSR Analytical Balance using distilled water as immersion liquid. The determinations were done at room temperature (RT) in triplicate and the averages reported (uncertainties in third decimal place). Other physical parameters deemed useful for characterizing the glasses were also calculated in accord with corresponding formulas.^7,18^ The average molar mass (*M*_av_) was calculated by
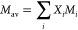
1where *X*_*i*_ and *M*_*i*_ are the mole fraction and molar mass of the *i*th component, respectively. From the measured densities (ρ), the molar volumes (*V*_m_) were obtained as
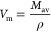
2The concentration (*N*) of Nd^3+^ ions in the glasses was calculated with the corresponding mole fractions (*X*), the glass densities, and the average molar masses according to
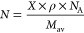
3where *N*_A_ is Avogadro’s constant. The mean interionic distances (*d*_Nd–Nd_) between Nd^3+^ ions were then calculated from the following relation
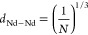
4Then, the average phosphorus–phosphorus distances (*d*_P–P_) connected with a glass structure were calculated by^7^
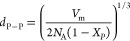
5where *X*_P_ is the mole fraction of P_2_O_5_ and *N*_A_ is Avogadro’s constant.

#### 2.2.2 XRD

Powder XRD was performed to verify the amorphous nature of the glasses (crushed to powder by mortar and pestle) with a PANalytical Empyrean X-ray diffractometer operating at RT using the available source with Mo-Kα radiation (λ = 0.71 Å). The acceleration voltage and current used were 60 kV and 40 mA, respectively.

#### 2.2.3 Raman Spectroscopy

Raman spectra were recorded at RT using the polished glass slabs with a Thermo Scientific DXR Raman microscope operating at 532 nm and a power of 10 mW. A 10× MPlan objective was employed for data collection with the acquisition time for each spectrum set at 100 s. Baseline subtraction was done using OriginPro, after which the spectra were normalized for comparison.

#### 2.2.4 XPS

XPS measurements were carried out on the glass slabs at RT using a Thermo K α XPS system with monochromatic Al-Kα X-ray source (1486 eV). A Flood gun (low energy ionized argon beam) was used to neutralize charging effects since samples are nonconductive. Samples were sputter-cleaned with an argon ion beam for 30 s to remove surface contaminants before collecting data. The adventitious carbon (C 1s) peak at 284.8 eV was used as an internal reference for peak position determinations. The binding energies for individual O 1s and P 2p contributions were determined by fitting procedures using Gaussian–Lorentzian peak shapes.

#### 2.2.5 DSC

Calorimetric analysis was carried out for the various glasses in a SDT650 calorimeter (TA Instruments) in alumina pans using a heating rate of 10 °C/min and N_2_ gas atmosphere with a flow rate of 100 mL/min. The thermal parameters of glass transition temperature (*T*_g_), onset of crystallization (*T*_x_), peak crystallization temperature (*T*_c_), and specific enthalpy change of crystallization (Δ*h*_c_) were then determined (TRIOS instrument’s software). The midpoint-inflection approach was used for the determination of the *T*_g_ values, whereas the *T*_x_, *T*_c_, and Δ*h*_c_ parameters related to crystallization were obtained following integration of the crystallization exotherms.

#### 2.2.6 Dilatometry

Dilatometry measurements were carried out on the 2.54 cm long glass cylinders in an Orton dilatometer (Model 1410B) operating at a heating rate of 3 °C/min under an ambient atmosphere. The determination of the parameters of dilatometric or softening temperature (*T*_s_) and linear coefficient of thermal expansion (CTE) was then carried out through the instrument’s software.

#### 2.2.7 UV–Vis–NIR Spectrophotometry

UV–vis–NIR optical absorption measurements were performed at RT on the ∼1 mm thick glass samples fixed on a sample holder with an Agilent Cary 5000 double-beam spectrophotometer; the reference in the measurements was air.

#### 2.2.8 PL Spectroscopy

PL spectra were collected at RT under static conditions with a Horiba Fluorolog-QM spectrofluorometer equipped with a continuous illumination Xe lamp and an InGaAs detector. The Xe flash lamp (pulse width of ∼2 μs) of the instrument was employed for recording emission decay curves. The PL measurements were recorded with the glass samples mounted in a solid sample holder keeping the sample position and conditions constant during experiments.

## 3 Results and Discussion

### 3.1 Density and Basic Physical Properties

The densities measured along with other physical parameters calculated for the different glasses are presented in Table 2. The densities are observed to first decrease slightly for the 0.5Nd and 1Nd relative to the BP host, suggesting slightly higher molar volumes upon neodymium doping. Similar fluctuations in density have been reported by Ismail et al.^19^ for multicomponent phosphate glasses with 60P_2_O_5_-8Al_2_O_3_-2Na_2_O-17K_2_O-(13 – *x*)BaO-*x*Nd_2_O_3_ with *x* = 0, 0.5, 0.75, 1.0, and 1.5 compositions. The density of the BP glass of 3.700 g/cm^3^ is slightly higher than, yet reasonably close to the reported for an equivalent glass melted using an alumina crucible at 3.680 g/cm^3^.^18^ The densities for the Nd-containing glasses in Table 2 then increase steadily with Nd_2_O_3_ replacing BaO, analogous to the effect seen for europium substituting barium in the glass system.^18^ The trend of increasing density is also consistent with reports from other groups for different glass systems wherein the concentration of Nd_2_O_3_ is increased.^4,7,11^ The average molar masses in Table 2 increase steadily as anticipated, a trend also exhibited for the molar volumes throughout the entire glass set within the 39.90–40.99 cm^3^/mol range. With the increase in Nd_2_O_3_ contents in the 0.5–4Nd glasses, the Nd^3+^ concentrations vary within the 1.49–11.75 × 10^20^ ions/cm^3^ range. The values for the mean Nd^3+^–Nd^3+^ interionic distances then decrease significantly, namely, from 18.9 Å for 0.5 mol % Nd_2_O_3_ to 9.48 Å with 4 mol % Nd_2_O_3_. These values are within the reported by Sontakke et al.^4^ for glasses with (100 – *x*)(20.95BaO-11.72Al_2_O_3_-56.12P_2_O_5_-6.79SiO_2_-3.91B_2_O_3_-0.51Nb_2_O_5_) + *x*Nd_2_O_3_ compositions in the 33.10–9.09 Å range for 0.1 ≤ *x* ≤ 5.0 mol % Nd_2_O_3_. In addition, the P^5+^–P^5+^ mean distances in Table 2 generally increase from the BP through the 0.5–4Nd glasses in the 4.046–4.083 Å range. This suggests an impact from increasing Nd_2_O_3_ contents on the structure of the phosphate network.^7^ Lastly, the nominal [O]/[P] ratio changes from the metaphosphate in the 50P_2_O_5_-50BaO binary BP glass toward the polyphosphate type where [O]/[P] = 3.08 for the 4Nd glass. As shall be considered, this appears to affect the Raman spectra suggesting that a depolymerization effect is induced with increasing Nd_2_O_3_ content in the glasses.

**Table 2:** Parameters Related to the Basic Physical Properties of the Different Glasses

	glass					
parameter	BP	0.5Nd	1Nd	2Nd	3Nd	4Nd
density, ρ (g/cm^3^)	3.700	3.670	3.674	3.717	3.744	3.780
average molar mass, *M*_av_ (g/mol)	147.64	148.55	149.47	151.30	153.13	154.96
molar volume, *V*_m_ (cm^3^/mol)	39.90	40.48	40.68	40.70	40.90	40.99
Nd^3+^ concentration, *N* (× 10^20^ ions/cm^3^)		1.49	2.96	5.92	8.83	11.75
Nd^3+^–Nd^3+^ mean distance, *d*_Nd–Nd_ (Å)		18.9	15.0	11.9	10.4	9.48
P^5+^–P^5+^ mean distance, *d*_P–P_ (Å)	4.046	4.066	4.073	4.073	4.080	4.083
[O]/[P]	3.00	3.01	3.02	3.04	3.06	3.08

### 3.2 Structural Assessment

Shown in Figure 1 are the XRD patterns obtained for the BP and 0.5–4Nd glasses with the Mo-*K*_α_ radiation (the photograph presented in the inset shows the different glasses as slabs). The diffractograms present analogous humps due to diffuse scattering stemming from long-range structural disorder. These appear however shifted toward lower angles compared to the typically obtained when Cu-Kα radiation (1.54 Å) is used^16,18^ in connection with the shorter wavelength of Mo-Kα X-ray photons (0.71 Å). The diffractograms presented in Figure 1 still do not show distinct crystallization peaks. The amorphous nature of the 50P_2_O_5_-(50 – *x*)BaO-*x*Nd_2_O_3_ (*x* = 0, 0.5, 1.0, 2.0, 3.0, 4.0 mol %) glasses synthesized is thus supported.

**Figure 2:**
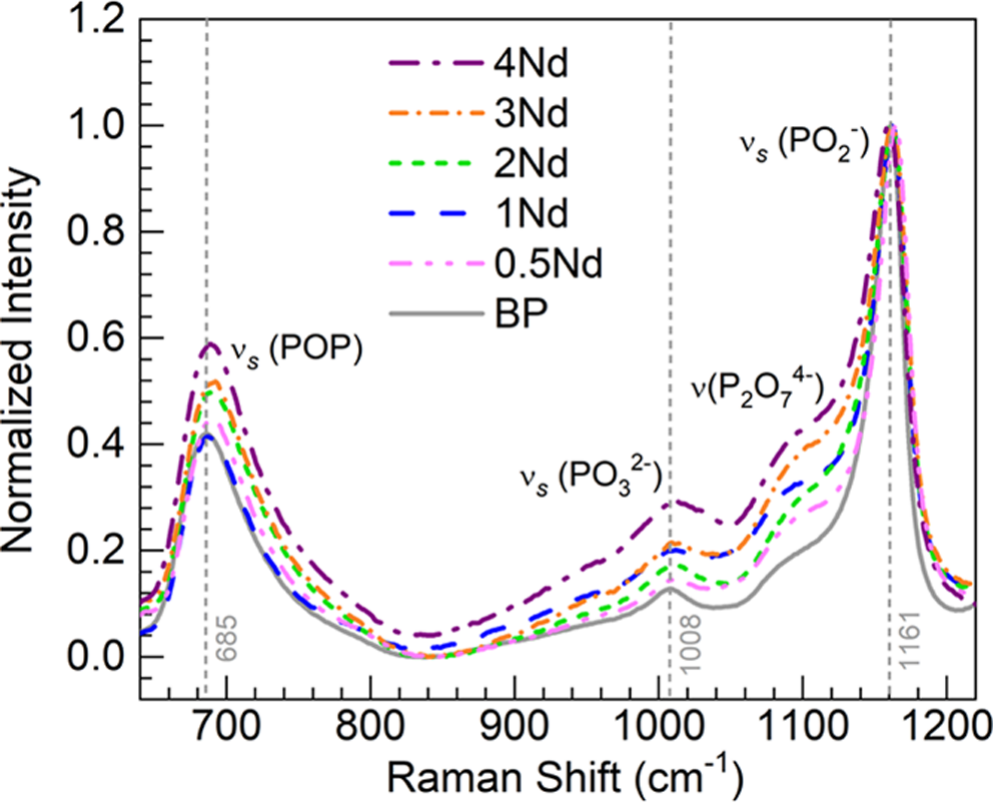
Normalized Raman spectra for the various glasses within the 640–1210 cm^–1^ spectral range for comparison; main spectroscopic features in the BP glass as a reference are indicated (vertical dashed lines–wavenumbers displayed).

Shown in Figure 2 are the Raman spectra obtained for the BP and 0.5–4Nd glasses. Following baseline subtraction, the spectra were normalized with respect to the strongest band within the 640–1120 cm^–1^ spectral range. The designation for the various features is accomplished in accord with reports in the literature for similar glasses.^5,14,18,20,21^ Considered as reference, the BP glass host presents toward the low energy region a band around 685 cm^–1^ credited to the in-chain symmetric stretching vibrations in P–O–P bridges, ν_s_(POP), in *Q*^2^ tetrahedral units (PO_4_ tetrahedra with 2 bridging oxygens, BOs). The small feature observed around 1008 cm^–1^ is ascribed to the symmetric stretch, ν_s_(PO_3_^2–^), in nonbridging oxygens (NBOs) pertaining to *Q*^1^ units (PO_4_ tetrahedra with 1 BO). Then, the binary BP glass shows the most intense band around 1161 cm^–1^ in connection with the out-of-chain symmetric stretch in PO_2_^–^ groups, ν_s_(PO_2_^–^), that come about in the NBOs of the *Q*^2^ units.

It is noticeable for the 0.5–4Nd glasses in Figure 2 that a feature manifests and intensifies toward the lower frequency wing of the ν_s_(PO_2_^–^) band around 1100 cm^–1^. This manifestation is indicative of stretching vibrations of *Q*^1^ units in P_2_O_7_^4–^ dimers^14,21,22^ which suggests that a chain length shortening effect in the phosphate network is promoted by Nd^3+^ ions inclusion. An increased intensity of the ν_s_(PO_3_^2–^) band is also noticeable for the Nd-containing glasses especially for the 4Nd glass. These considerations point to Nd^3+^ ions producing glass depolymerization relative to the BP host glass^14,18,19^ also harmonizing with the increasing [O]/[P] ratios (Table 2). Additionally, toward the low frequency region, the ν_s_(POP) band (BO-related) shows some differences among the glasses, for instance, concerning position, width, and intensity relative to the ν_s_(PO_2_^–^) band (NBO-related). This type of behavior has been associated with depolymerization effects in phosphate glasses.^18,20^ Namely, shorter PO_4_ tetrahedra chains yield higher frequency components of the ν_s_(POP) band, where an asymmetric extension toward higher frequencies points to asymmetric distributions of chain lengths toward shorter chains. The current appraisal is then in agreement with reports indicating that Nd^3+^ doping of phosphate-based glasses produced a depolymerization effect as probed by various spectroscopies including ^31^P nuclear magnetic resonance (NMR).^7,14,22−24^

**Figure 3:**
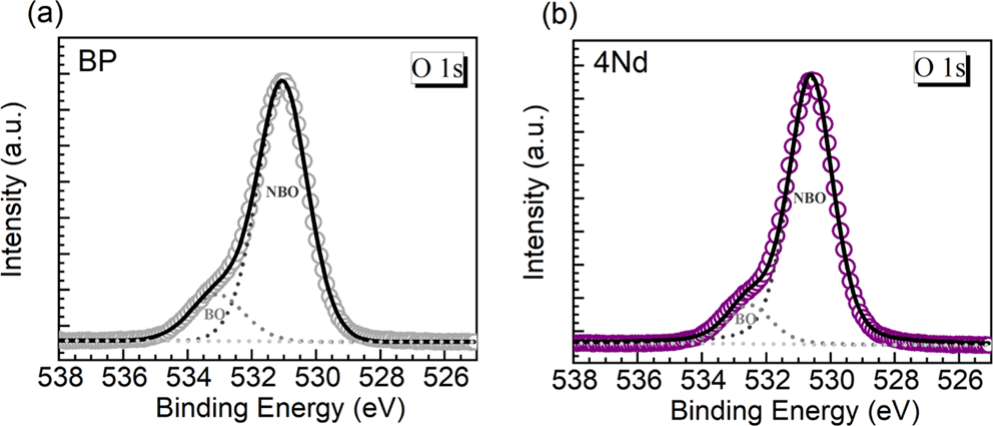
XPS O 1s peaks registered for (a) BP and (b) 4Nd glasses (open symbols) with the corresponding deconvolutions (results summarized in Table 3) into the different oxygen species (BO, bridging oxygen and NBO, nonbridging oxygen; dotted curves). The cumulative fits are the solid traces.

To corroborate that replacing BaO by Nd_2_O_3_ depolymerized the phosphate network, the oxygen bonding environment was examined by XPS for the BP and 4Nd glasses bracketing the concentration range at 0 and 4 mol % Nd_2_O_3_, respectively. Despite XPS being a surface analysis technique, following proper cleaning procedures, the surface of glass materials can be considered representative of the bulk.^25^ Specifically, O 1s XPS data allows for the distinction between BOs in the glass network and NBOs interacting with network modifiers; the different proportions of these can be then linked to the degree of polymerization.^25,26^ The O 1s XPS data obtained for the two glasses following the Ar^+^ sputtering for cleaning the surfaces is shown in Figure 3a,b. The experimental spectra displayed peaks at about 531.1 and 530.6 eV for the BP and Nd glasses, respectively. In addition, the spectra exhibit a shoulder toward the high BE side. This feature corresponds to BOs (P–O–P) in the structural network, while the dominant peak at lower BE reflects the presence of terminal NBOs (P–O^–^) interacting with metal cations.^25,26^ Hence, the spectra were deconvoluted into the two oxygen contributions, and the resulting bands are also presented overlaid with the experimental traces and the cumulative fits in each panel in Figure 3. The corresponding parameters of binding energy (BE), full width at half-maximum (FWHM), and % relative area are summarized in Table 3. The BEs for the 4Nd glass appear shifted toward lower energy while the components exhibited a band narrowing effect relative to the BP host. This can be associated with a more prominent presence of NBOs in the 4Nd glass. In fact, as a key parameter deduced from the areas of the two bands, the relative amounts of NBOs were estimated at 83.6 and 87.3% for the BP and Nd glasses, respectively. The NBO content clearly increases with the exchange of the 4 mol % BaO with Nd_2_O_3_, thus implying that depolymerization was induced. Therefore, the O 1s XPS data supports the Raman spectra interpretations of shorter chain lengths and wider distributions produced in the phosphate networks with increasing Nd_2_O_3_ contents.

**Table 3:** O 1s Binding Energy (BE), Full Width at Half-Maximum (FWHM), and % Relative Area for the Different Oxygen (BO, Bridging Oxygen; NBO, Nonbridging Oxygen) Components for the BP and 4Nd Glasses as Estimated from Decomposing the XPS Spectra (Figure 3)

		O 1s XPS		
glass	component	BE (eV)	FWHM (eV)	% area
BP	BO	533.1	2.0	16.4
	NBO	531.1	1.8	83.6
4Nd	BO	532.5	1.6	12.7
	NBO	530.6	1.6	87.3

**Figure 4:**
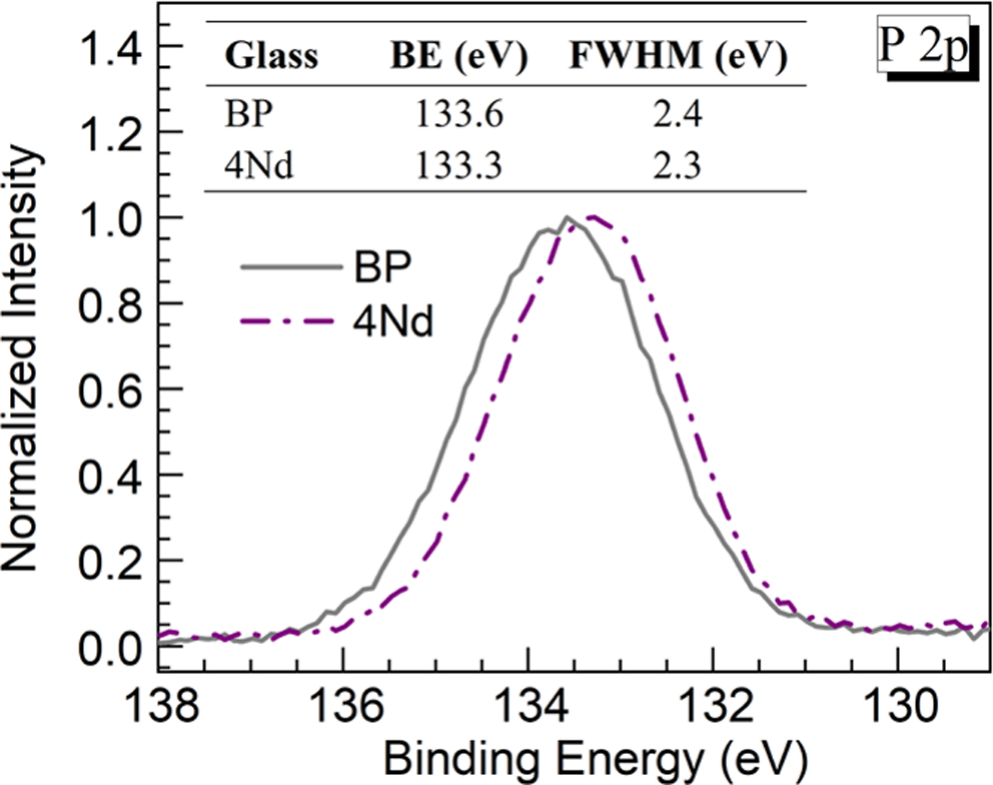
Normalized XPS P 2p peaks for the BP and 4Nd glasses. The values of peak binding energy (BE) and full width at half-maximum (FWHM) for each glass are presented in the table embedded.

Additional information may be obtained through the P 2p peaks registered for the BP and 4Nd glasses shown in Figure 4. The corresponding peak BE and FWHM values are presented in the embedded table. It is observed that the BE for the P 2p peak of the 4Nd glass (133.3 eV) is shifted to a lower value relative to the BP host (133.6 eV) which also appears somewhat broader. The BE of P 2p electrons is known to be sensitive to the charge associated with the phosphorus atoms as shown by Pelavin et al.^27^ in their work on various phosphorus-containing compounds. With regards to glasses, Gresch et al.^28^ performed XPS characterization of sodium phosphate glasses also focusing on BE shifts of the P 2p and P 2s peaks. The authors noticed that the BE of the peaks decreased as the content of Na_2_O as a network modifier increased.^28^ Such manifestation may be linked with a larger number of NBO (P–O^–^) increasing electron density toward phosphorus. The present result for the BE of the 4Nd glass (133.3 eV) being shifted to lower energy compared to the BP glass (133.6 eV) agrees with this interpretation since the former glass also showed a higher NBO content (Table 3). Accordingly, both the O 1s and P 2p XPS results examined herein are consistent with the Raman spectra indicating glass depolymerization being induced while Nd_2_O_3_ is added at the expense of BaO. Having thus set the background with respect to the basic physical and structural properties, the results from thermal analysis are considered next.

### 3.3 Thermal Analyses

**Figure 5:**
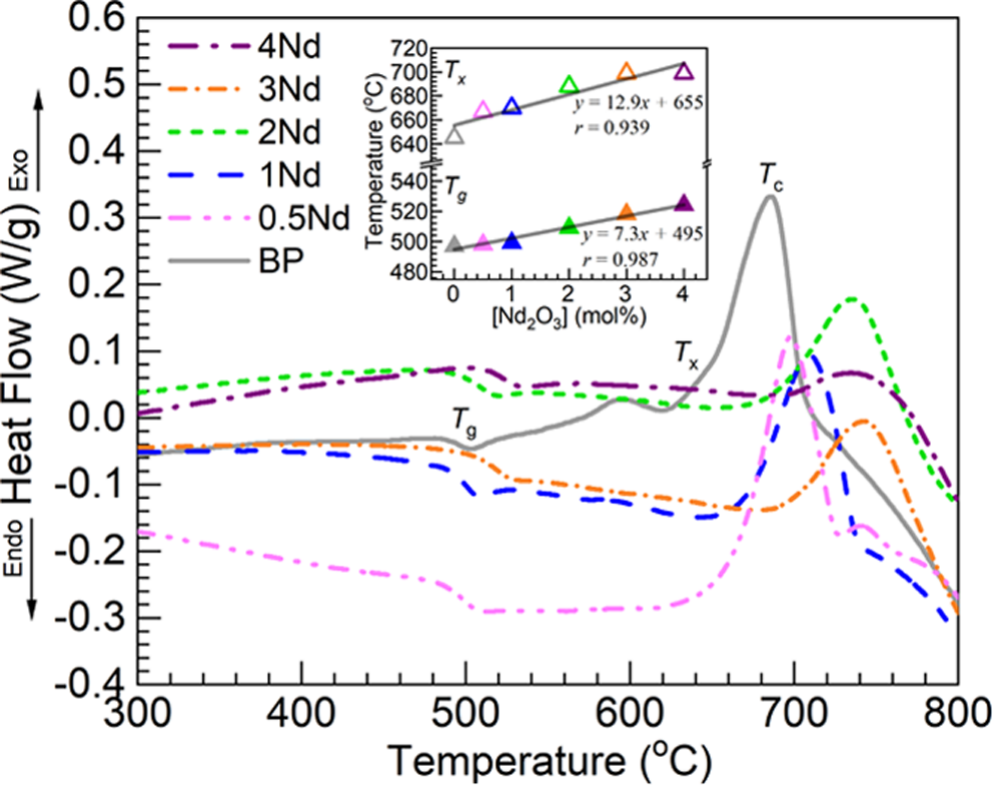
DSC profiles obtained for the different glasses displaying the regions of glass transition (*T*_g_), onset of crystallization (*T*_x_), and crystallization temperature (*T*_c_); estimated values are presented in Table 4. The inset is a plot of the *T*_g_ (filled triangles) and *T*_x_ (open triangles) values estimated vs Nd_2_O_3_ concentration in the glasses; the solid lines are linear fits to the data (equations and correlation coefficients, *r*, displayed).

Presented in Figure 5 are the DSC profiles obtained for the BP and 0.5–4Nd glasses within the 300–800 °C range. From these, the peak crystallization temperatures, *T*_c_, the onset of crystallization, *T*_x_, and glass transition temperatures, *T*_g_, were determined. The corresponding values estimated are summarized in Table 4. Also shown in Table 4 are (i) the additional parameter, Δ*T = T*_x_ – *T*_g_, indicating glass stability^29^ and (ii) the specific enthalpy change of crystallization, Δ*h*_c_, calculated following integration of the crystallization peaks. The BP glass host taken as a reference presents a main crystallization peak around 686 °C with onset temperature at 645 °C. Then, within the glass transition region, the estimated value for the *T*_g_ was 497 °C which is in good agreement with the reported for an equivalent glass melted using an alumina crucible at 494 °C.^18^ The implied thermal stability factor was then Δ*T =* 148 °C, and Δ*h*_c_ = −81 kJ/kg. An interesting development is observed in Table 4 for the various parameters with increasing Nd_2_O_3_ added at the expense of BaO in the glasses. The *T*_g_ values increase throughout within 498–524 °C with increasing Nd^3+^ concentration in the 0.5–4Nd glasses in a similar fashion to the reported for europium substituting barium in the glass system.^18^ These can be considered on the high side compared to the values reported (367–485 °C) for different commercially available Nd laser glasses having multiple components some of which tend to decrease the *T*_g_.^2^ The *T*_g_ values obtained herein were further plotted in the inset of Figure 5 as a function of the Nd_2_O_3_ concentration in the glasses. Regression analysis yielded a correlation coefficient *r* of 0.987 indicating good linearity, with an intercept of 495 °C close to the measured *T*_g_ of the BP glass of 497 °C (Table 4). The upward trend in *T*_g_ in the present work is indicative of a glass strengthening effect despite the depolymerization induced by the Nd^3+^ ions (*vide supra*). In this regard, we consider the work by Sendova et al.^30^ on lanthanide-doped phosphate glasses by DSC. The study which included neodymium among others revealed a correlation of the glass transition activation energy with the trivalent lanthanide radius.^30^ Along this line, increasing trends in the *T*_g_ have been reported by Wang et al.^31^ for calcium phosphate glasses with 50P_2_O_5_-(50 – *x*)CaO-*x*Gd_2_O_3_ (0 ≤ *x* ≤ 6 mol %) compositions and by Xu et al.^32^ for 60P_2_O_5_–25 Bi_2_O_3_-(10 – *x*)CaO-5Sb_2_O_3_-*x*Gd_2_O_3_ (*x* = 0, 1, 2, 3, 4 mol %) glasses assessed by dilatometry. It has been discussed by the authors^31,32^ that the higher ionic field strength of the lanthanide ions compared to the alkaline earth metal is at the origin of the thermal behavior. Underpinning the impact of cation field strength, Hayden et al.,^1^ Campbell,^2^ and Campbell and Suratwala^3^ reported broadly on the effect of various alkali/alkaline earth cations in neodymium laser glasses on various properties including thermomechanical attributes. For the assessment in the present investigation, we may then consider the ionic radii of Ba^2+^ and Nd^3+^ cations reported by Shannon^33^ of 1.42 and 0.983 Å for Ba^2+^ and Nd^3+^ cations, respectively, for anticipated coordination numbers of eight for Ba^2+^^34^ and six for Nd^3+^.^35^ The ionic field strengths (*F*) may be then calculated as
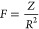
6where *Z* is the cation charge and *R* is the ionic radius. The resulting *F* values are 0.992 and 3.105 Å^–2^ for Ba^2+^ and Nd^3+^, respectively. Thus, replacing Ba^2+^ ions with an increasing amount of high-field strength Nd^3+^ ions in the 50P_2_O_5_-(50 – *x*)BaO-*x*Nd_2_O_3_ glass system under study is likely conducing to a bond strengthening effect on NBOs in the phosphate network.

**Table 4:** Glass Transition Temperature (*T*_g_, Estimated from Midpoint-Inflection Approach), Onset of Cystallization (*T*_x_), Main Peak Crystallization (*T*_c_) Temperature, Thermal Stability Parameter Δ*T = T*_x_ – *T*_g_, and Specific Enthalpy of Crystallization (Δ*h*_c_) Estimated for the Various Glasses from the DSC Profiles

glass	*T*_g_ (°C)	*T*_x_ (°C)	*T*_c_ (°C)	Δ*T = T*_x_ – *T*_g_ (°C)	Δ*h*_c_ (kJ/kg)
BP	497	645	686	148	–81
0.5Nd	498	667	698	169	–63
1Nd	499	670	710	171	–70
2Nd	509	688	736	179	–56
3Nd	518	699	744	181	–48
4Nd	524	699	735	175	–11

As also perceived from Table 4, a general trend toward higher temperatures takes place for the *T*_x_ values with increasing Nd_2_O_3_ contents. The values are also plotted together with the *T*_g_ in the inset of Figure 5 as a function of the Nd_2_O_3_ concentration in the glasses. Regression analysis yielded a correlation coefficient *r* of 0.939 indicating less linearity compared to the *T*_g_. The intercept of 655 °C is also further apart from the *T*_x_ value for the BP host of 645 °C (Table 4). The increasing *T*_x_ behavior also concurs with the report by Wang et al.^31^ for the gadolinium-loaded calcium phosphate glasses pointing to thermal stability improvements. In addition, we notice a trend for the peak crystallization temperatures to increase up to the 3Nd glass but then decrease somewhat for the 4Nd glass. Then, the Δ*T = T*_x_ – *T*_g_ thermal stability parameter behaves in like manner. However, the crystallization exotherms in Figure 5 become in general suppressed with increasing Nd^3+^ concentration. This translated into an overall decreasing trend of the magnitudes of the specific enthalpy change of crystallization (Table 4). The DSC data thus supports a suppression of glass crystallization with increasing Nd_2_O_3_ content as the process appears with less exothermic character. It then overall appears that the inclusion of Nd^3+^ ions leads to improved thermal properties and enhanced glass stability. Seeking then to complement the thermal characterization, results from dilatometry measurements are considered next.

**Figure 6:**
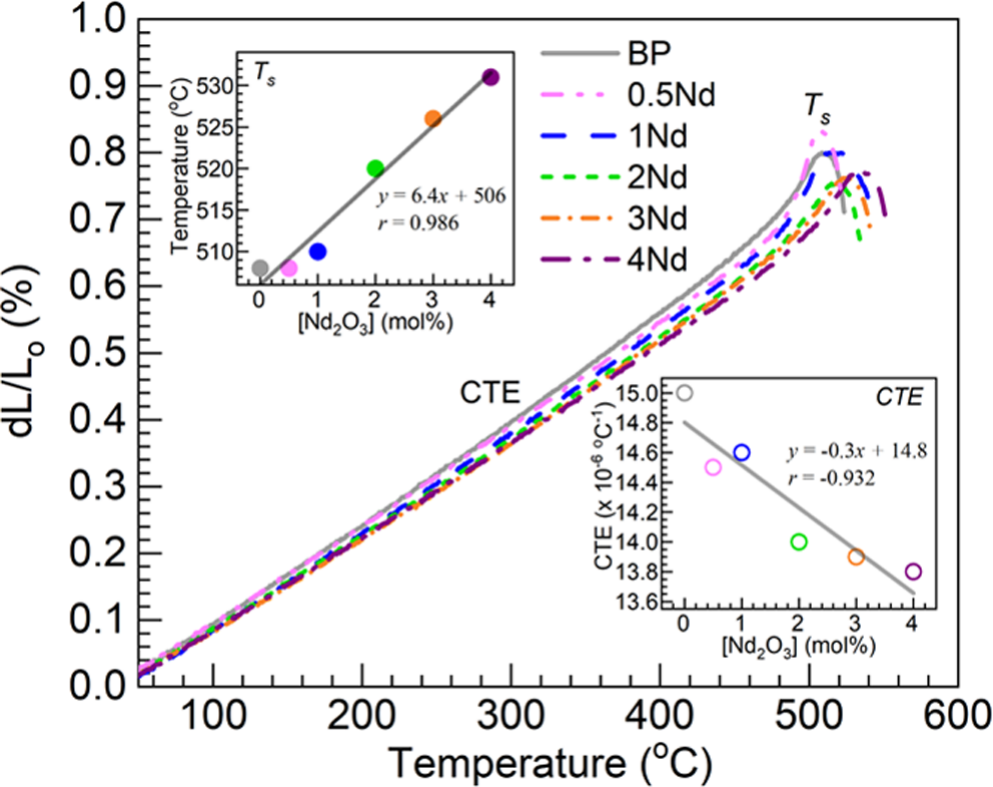
Dilatometric profiles obtained for the different glasses; estimated values of coefficient of thermal expansion (CTE) and softening temperature (*T*_s_) presented in Table 5. The top and bottom insets are plots of the *T*_s_ (filled circles) and CTE (open circles) values estimated, respectively, vs Nd_2_O_3_ concentration in the glasses; the solid lines are linear fits to the data (equations and correlation coefficients, *r*, displayed).

Figure 6 shows the dilatometric profiles obtained for the BP and 0.5–4Nd glasses under consideration. Beginning with the BP glass as the reference, the evolution of the linear expansion profiles *dL*/*L*_o_ (%) vs temperature were used for the extraction of the parameters of coefficient of thermal expansion, CTE, and dilatometric or softening temperature, *T*_s_.^18,36^ The linear CTE values were determined in the 50–400 °C range consistently, whereas *T*_s_ is the peak temperature within the expansion region (since the *T*_g_ values were determined by DSC above, the estimation of such by dilatometry was not pursued). The different CTE and *T*_s_ values obtained are summarized in Table 5; these are also plotted in the insets of Figure 6 as a function of the Nd_2_O_3_ concentration in the glasses. The *T*_s_ and CTE values for the BP reference glass of 508 °C and 15.0 × 10^–6^ °C^–1^, respectively, are in reasonable agreement with the reported values of 511 °C and 14.9 × 10^–6^ °C^–1^ for an equivalent glass prepared using a high purity alumina crucible.^18^ The *T*_s_ values were then estimated at 508 °C for both the BP and 0.5Nd glasses, but are then observed in Table 5 to increase throughout for the 0.5–4Nd glass set. This latter tendency concurs with the upward trend in the *T*_g_ values obtained from DSC (Table 4) pointing to a glass strengthening effect induced by Nd^3+^ ions having higher field strength than Ba^2+^. Regression analysis performed on the *T*_s_ values in the top inset of Figure 6 yielded a correlation coefficient *r* of 0.986 indicating reasonable correlation, with an intercept of 506 °C close to the *T*_s_ measured for the BP host at 508 °C (Table 5). The slope of 6.4 °C/mol % is also reasonably close to the one obtained for the *T*_g_ of 7.3 °C/mol % (Figure 5, inset).

**Table 5:** Values of Coefficient of Linear Thermal Expansion (CTE, Estimated in the 50–400 °C Range) and Dilatometric or Softening Temperature (*T*_s_) Obtained for the Different Glasses from Dilatometry

glass	CTE (×10^–6^ °C^–1^)	*T*_s_ (°C)
BP	15.0	508
0.5Nd	14.5	508
1Nd	14.6	510
2Nd	14.0	520
3Nd	13.9	526
4Nd	13.8	531

Further, the CTE values in Table 5 generally decrease with increasing Nd_2_O_3_ contents, namely, from 15.0 × 10^–6^ °C^–1^ in the BP glass host to 13.8 × 10^–6^ °C^–1^ in the 4Nd glass. The value of 14.5 × 10^–6^ °C^–1^ for the 0.5Nd glass already decreased considerably relative to the BP reference. The CTE of the 1Nd glass of 14.6 × 10^–6^ °C^–1^ is however comparable to the 0.5Nd glass considering that the expected error is within ±0.1 × 10^–6^ °C^–1^.^36^ The CTE of the 0.5–4Nd glasses can be considered to be on the high side compared to the values reported for commercial laser glasses having multiple components some of which tend to decrease the CTE.^2,5^ However, the value for the 4Nd glass of 13.8 × 10^–6^ °C^–1^ is close to the reported within the 20–300 °C range for Schott’s LG-770 high-energy/high-power (HEHP) laser glass of 13.4 × 10^–6^ °C^–1^ which is of the aluminophosphate type.^2^ Moreover, the 4Nd glass herein has significantly lower CTE than the reported for the phosphate laser glass made by Muñoz-Quiñonero et al.^10^ with 2 wt % Nd_2_O_3_ estimated at 17.7 × 10^–6^ °C^–1^ within the 20–300 °C range. The regression analysis for the CTE values as shown in the bottom inset of Figure 6 did not exhibit as good a linear correlation coefficient (*r* = −0.932) as the *T*_s_ (*r* = 0.986). Still, the obtained intercept from the linear fit of 14.8 × 10^–6^ °C^–1^ is close to the CTE measured for the BP glass of 15.0 × 10^–6^ °C^–1^ (Table 5).

In their dilatometric evaluation of the glasses with 60P_2_O_5_–25 Bi_2_O_3_-(10 – *x*)CaO-5Sb_2_O_3_-*x*Gd_2_O_3_ (*x* = 0, 1, 2, 3, 4 mol %) compositions, Xu et al.^32^ also reported increasing softening temperatures (456–476 °C range) and decreased CTE values (9.08 × 10^–6^ – 8.76 × 10^–6^ °C^–1^ range). The authors interpreted the trends in terms of the ionic field strength of Gd^3+^.^32^ Such an argument is consistent with the report by Hayden et al.^1^ presenting a significant correlation for the CTE with the average field strength of the alkali and alkaline earth cations in laser glass. As also pointed out by Li et al.^5^ on their investigation on thermal properties of Nd-doped phosphate glass, the increase in cation field strength is expected to lead to a tighter network conducing to a lower CTE. It is then likely that the decrease in CTE of the glasses studied in this work takes place following the increasing concentration of high-field strength Nd^3+^ ions (*F* = 3.105 Å^–2^) replacing Ba^2+^ ions with a lower value (*F* = 0.992 Å^–2^). It is also worth noting at this point that the CTE/*T*_*s*_ values in Table 5 for the 1Nd, 2Nd, and 4Nd glasses tend to be higher/lower than the obtained for glasses with an equivalent amount of europium oxide having 50P_2_O_5_-(50 – *x*)BaO-*x*Eu_2_O_3_ for *x* = 1, 2, and 4 mol % compositions reported at 14.8 × 10^–6^ °C^–1^/511 °C, 14.3 × 10^–6^ °C^–1^/526 °C, and 13.7 × 10^–6^ °C^–1^/538 °C, respectively.^18^ Here, we consider that for Eu^3+^, the ionic radius assuming 6-fold coordination^33^ is *R* = 0.947 Å which is smaller than the radius of Nd^3+^ (*R* = 0.983 Å), thus resulting in a field strength of 3.345 Å^–2^ calculated by eq 6. Hence, it is suggested that the lower field strength of Nd^3+^ (*F* = 3.105 Å^–2^) makes the glass structure less tight and more prone to thermal expansion than an equivalent amount of Eu^3+^ ions with higher field strength (*F* = 3.345 Å^–2^).

The fact that the CTE overall tends to decrease in the Nd-doped glasses as shown in Figure 6 and Table 5 is interesting in view of the depolymerization induced by Nd^3+^ ions as indicated by Raman spectroscopy and XPS results (*vide supra*). The reason for this assertion is that the changes in CTE can be dictated by opposing structural effects, namely, an increase in NBOs which increases CTE vs the increase in cation field strength which decreases the CTE. If the structural depolymerization was dominant, the high degree of disorder brought by a larger number of shorter PO_4_ tetrahedra chains would have been leading toward higher CTE. For instance, increasing CTE values were reported for bismuth borate glasses of (25 + *x*)Bi_2_O_3_–15BaO-10Li_2_O-(50 – *x*)B_2_O_3_ (*x* = 0, 10, 20, 30 mol %) compositions where the content of NBO also increased.^36^ Similarly, in their work on 10CaF_2_-(29.5–0.4*x*)CaO-(60–0.6*x*)B_2_O_3_-*x*TeO_2_-0.5Yb_2_O_3_ (*x* = 10, 16, 22, 31, and 54 mol %) glasses, de Oliveira Lima^37^ noticed that the CTE increased with TeO_2_ content which was also discussed in terms of NBOs producing loose network connectivity. In the present case then, the field strength effect dominates thus leading to overall reduced CTE values with Nd_2_O_3_ concentration regardless of the depolymerization. Henceforth, we follow with the optical spectroscopy evaluation centering on the luminescent properties of the NIR-emitting Nd^3+^ ions.

### 3.4 Optical Properties

**Figure 7:**
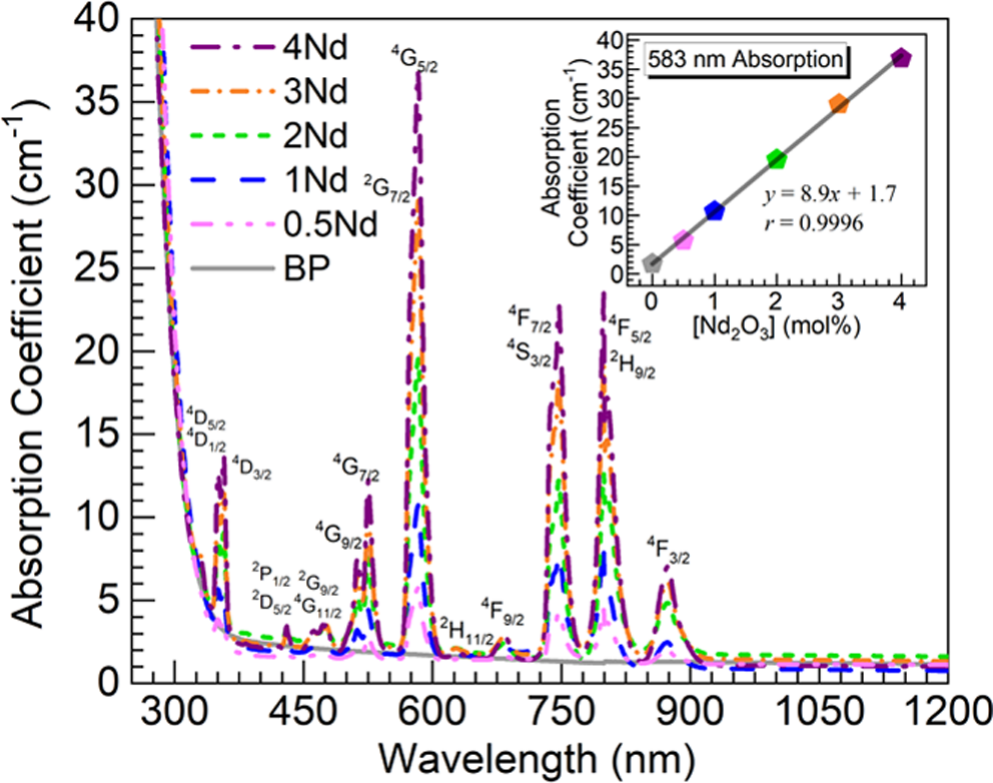
UV–vis–NIR absorption spectra for the different glasses. The inset is a plot of the absorption intensity at 583 nm vs Nd_2_O_3_ concentration in the glasses; the solid line is linear fit to the data (equation and correlation coefficient, *r*, displayed).

The UV–vis–NIR optical absorption spectra obtained for the various glasses under consideration are shown in Figure 7. While the undoped BP glass exhibits merely baseline absorption with the intrinsic rise in the UV edge, the 0.5–4Nd glasses display the different transitions spanning from the NIR to the visible range.^4,9−11^ Most prominently seen is the absorption peak around 583 nm in connection with ^4^*I*_9/2_ → ^4^*G*_5/2_ + ^2^*G*_7/2_ transitions in Nd^3+^ ions.^4,10^ The Nd^3+^ absorption intensity is observed to increase continuously with Nd_2_O_3_ content in the 0.5–4Nd glasses. An appraisal of this is made in the inset of Figure 7 where the peak intensity of the 583 nm absorption is plotted as a function of the Nd_2_O_3_ concentration in the glasses. The regression analysis performed on the data yielded a correlation coefficient *r* of 0.9996, thus indicating a strong linear correlation. It is then supported that the increasing amounts of Nd^3+^ ions were successfully incorporated in the 0.5–4Nd glasses.

**Figure 8:**
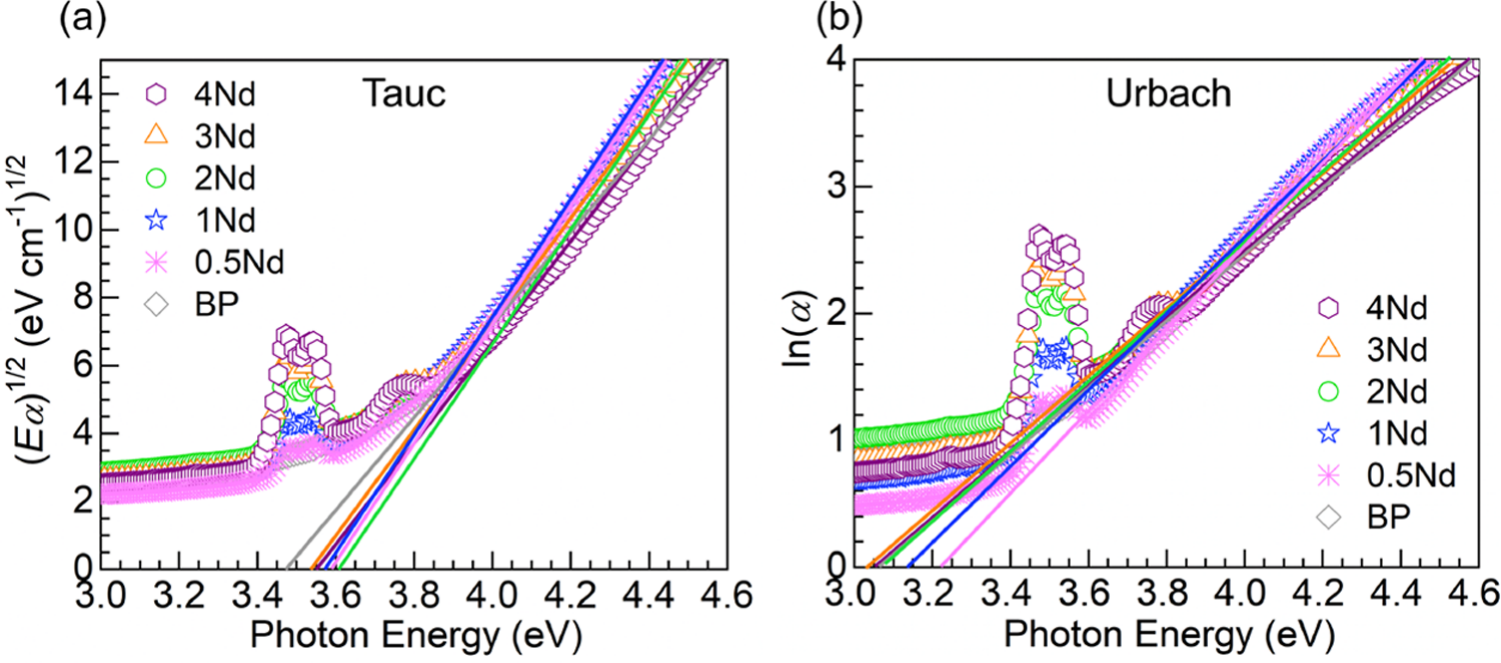
(a) Tauc plots (indirect band gap) and (b) Urbach plots obtained for the various glasses. The solid lines represent the linear regressions to the data from where the optical band gaps (*E*_opt_) and Urbach energies (*E*_U_) were estimated (values presented in Table 6).

The UV absorption edge of the glasses may be further examined in the context of Tauc and Urbach plots.^11,16,36,38−40^ The following expression for the absorption coefficient, α, as a function of photon energy (*hν*) may be utilized to estimate the optical band gap energy, *E*_opt_
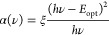
7where the exponent of 2 is associated with allowed indirect transitions anticipated for glasses and ξ is a constant.^36,40^ The corresponding Tauc plots of (*Eα*)^1/2^ vs photon energy (*hν*) generated are shown in Figure 8a, which were used to estimate *E*_opt_ from extrapolation of the linear portion to obtain the intercept on the energy axis. The *E*_opt_ values determined are presented in Table 6. The optical band gap energy of the 0.5Nd glass estimated at 3.59 (±0.05) eV is noticed to be higher than the BP host value of 3.47 (±0.03) eV. The remainder 1–4Nd glasses show some fluctuation in *E*_opt_; however, the values are still higher than the BP host. The recent work by Singh et al.^40^ on 35.4B_2_O_3_-20P_2_O_5_-34PbO-10Li_2_O-0.5Yb_2_O_3_ containing increasing amounts of Nd_2_O_3_ also showed a trend of increasing band gap energies. Nonetheless, the authors considered the results to be linked with an increased BO content,^40^ which is not the case in the present work. On the other hand, an opposing trend was reported by Algradee et al.^7^ for glasses with 20Li_2_O–40ZnO–40P_2_O_5_:*x*Nd_2_O_3_ molar composition with *x* = 0, 1, 2, 4, 6, 8 wt % wherein the optical band gap energies decreased with increasing Nd_2_O_3_ concentration. The authors interpreted the results in terms of a greater number of NBOs in the glasses.^7^ The present work is similar in the sense that a depolymerization effect was induced by Nd_2_O_3_ as supported by Raman and XPS (*vide supra*). Nevertheless, it contrasts in the sense that the band gap values herein tended to be higher than the undoped matrix. We may consider in the present work that the high-field strength Nd^3+^ ions (*F* = 3.105 Å^–2^) are replacing Ba^2+^ ions with lower field strength (*F* = 0.992 Å^–2^). It may be then suggested that the Nd^3+^ ions exert an influence by withdrawing electron density likely lowering the top of the valence band which can widen the band gap. The fluctuations observed for the 0.5–4Nd glasses in Table 6 may then reflect the influence of the absorption of Nd^3+^ ions toward the low energy side of the absorption edge presenting some interference [see Figure 8a].

**Table 6:** Estimated Parameters of Indirect Optical Band Gaps (*E*_opt_) and Urbach Energy (*E*_U_) for the Various Glasses

glass	*E*_opt_ (eV)	*E*_U_ (eV)
BP	3.47 (±0.03)	0.380 (±0.003)
0.5Nd	3.59 (±0.05)	0.310 (±0.003)
1Nd	3.57 (±0.05)	0.333 (±0.003)
2Nd	3.61 (±0.04)	0.368 (±0.004)
3Nd	3.54 (±0.06)	0.374 (±0.004)
4Nd	3.55 (±0.04)	0.377 (±0.004)

The Urbach energy, *E*_U_, associated with band tailing reflecting defects/disorder may be evaluated next from the following relation
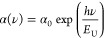
8where α_0_ is a constant and *hν* is the photon energy.^7,11,39^ Accordingly, the Urbach energy of the studied glasses was evaluated from the ln(α) vs *hν* plot as shown in Figure 8b. The *E*_U_ values determined are presented together with the indirect optical band gaps in Table 6. It is observed that the *E*_U_ value for the 0.5Nd glass at 0.310 (±0.003) eV decreased relative to the undoped BP glass with *E*_U_ of 0.380 (±0.003) eV. This suggests an increased ordering at the low Nd_2_O_3_ concentration of 0.5 mol % replacing an equal amount of BaO. Conversely, the Urbach energies for the 1–4Nd glasses exhibited an increasing trend, where the value for the 4Nd glass of 0.377 (±0.004) eV was comparable to the BP host. Algradee et al.^7^ observed in their work on glasses with 20Li_2_O–40ZnO–40P_2_O_5_:*x*Nd_2_O_3_ molar composition with *x* = 0, 1, 2, 4, 6, 8 wt % that the Urbach energies increased with Nd_2_O_3_ content. The authors attributed this behavior to a higher degree of structural disorder increasing the localized states in the gap.^7^ The increasing *E*_U_ values estimated herein for the Nd-containing glasses (Table 6) also point toward an increased disorder realized with increasing Nd_2_O_3_ content. This at least harmonizes with the Raman and XPS evaluation supporting high degree of depolymerization at high Nd^3+^ concentrations.

**Figure 9:**
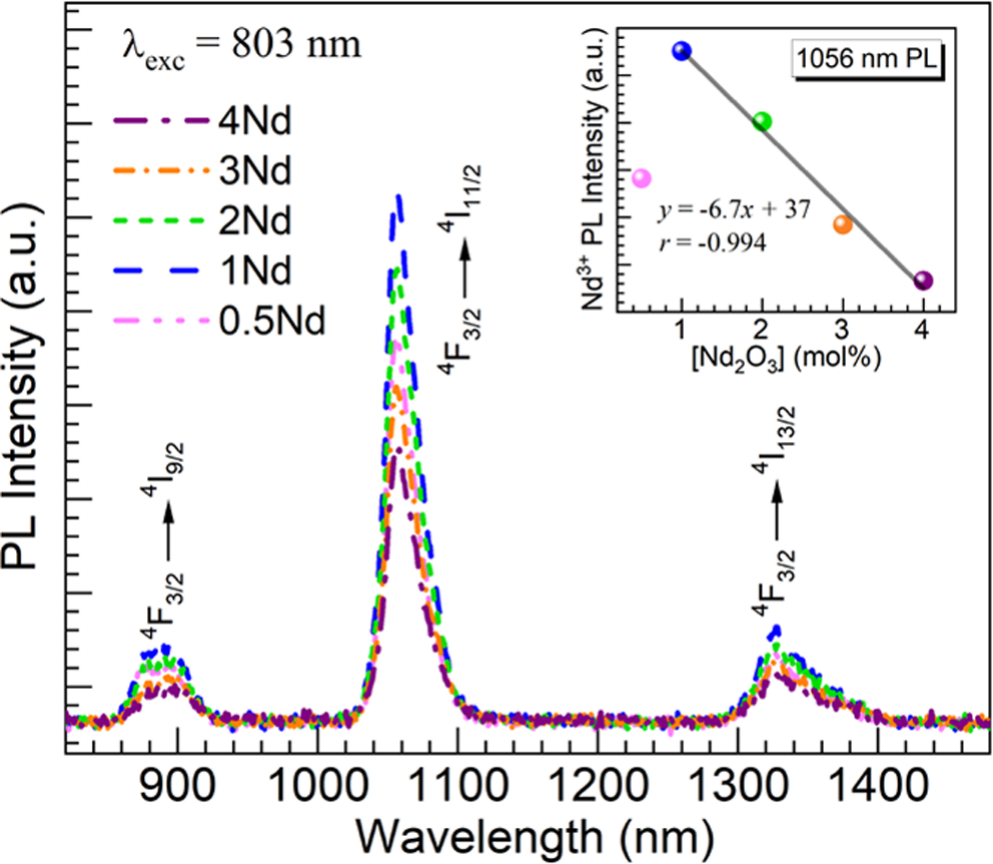
PL spectra obtained for the 0.5–4Nd glasses under excitation at 803 nm. The inset is a plot of the Nd^3+^ emission intensity at 1056 nm vs. Nd_2_O_3_ concentration in the glasses; the solid line is linear fit to the data for the 1–4Nd glasses (equation and correlation coefficient, *r*, displayed).

**Figure 10:**
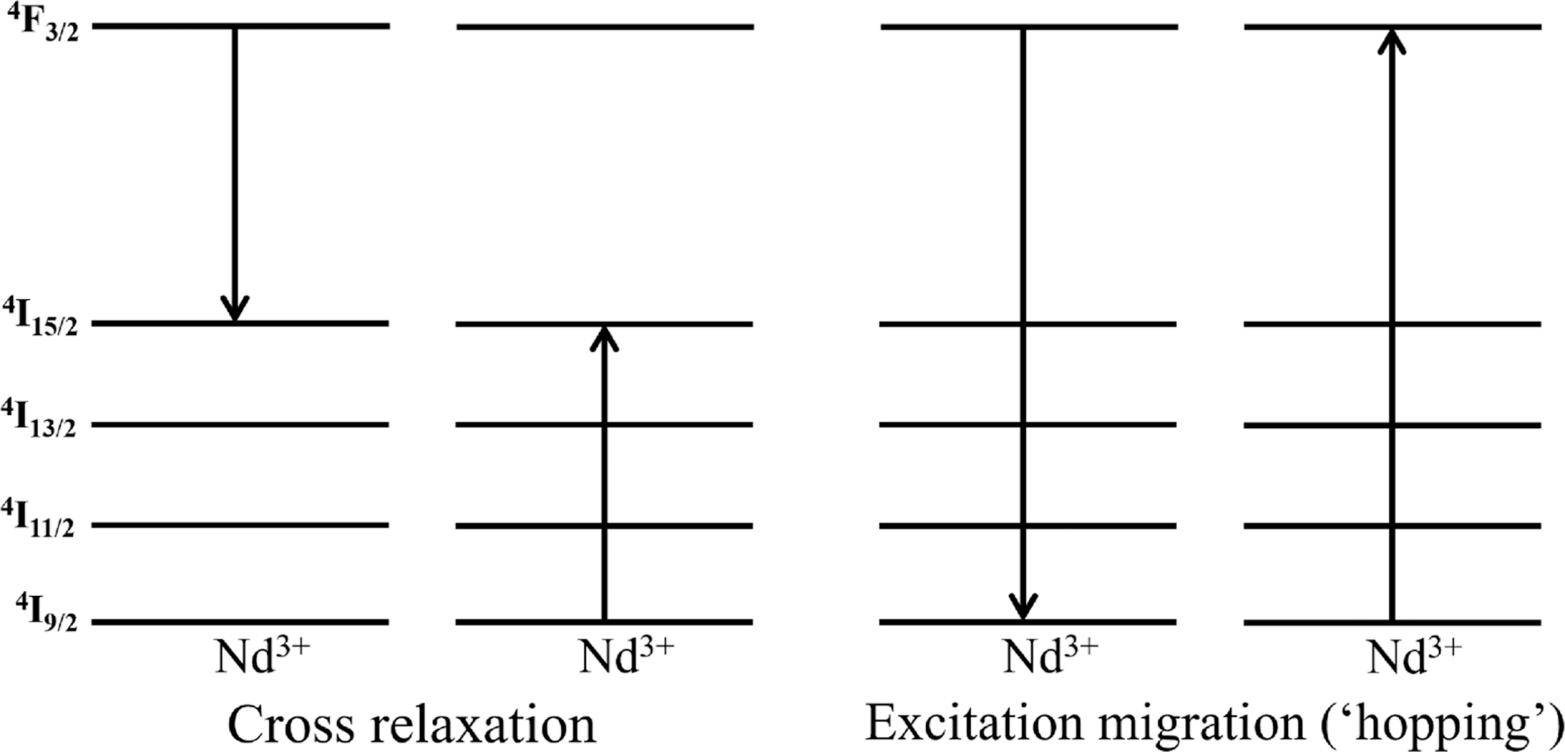
Representation of Nd^3+^–Nd^3+^ nonradiative interactions in laser phosphate glasses accountable for the PL concentration quenching effect.

Moving next to evaluating the PL properties, shown in Figure 9 are the NIR emission spectra obtained for the 0.5–4Nd glasses under excitation at 803 nm accessing ^4^*I*_9/2_ → ^4^*F*_5/2_ + ^2^*H*_9/2_ transitions in Nd^3+^ ions of practical values (being typically pumped with a diode source^2^). The emission spectra display the typical Nd^3+4^*F*_3/2_ → ^4^*I*_9/2_, ^4^*I*_11/2_, ^4^*I*_13/2_ NIR transitions^4,9,14,19,41^ observed around 890, 1056, and 1330 nm, respectively. While the ^4^*F*_3/2_ → ^4^*I*_9/2_, ^4^*I*_11/2_ emissions around 0.9 and 1.06 μm have been attractive for solar spectral conversion and lasing,^2,8,12,14^ the ^4^*F*_3/2_ → ^4^*I*_11/2_, ^4^*I*_13/2_ around 1.06 and 1.33 μm have been also considered interesting for telecommunications and medical applications.^42^ The evolution of the PL intensities is not like the absorption which increased linearly with Nd_2_O_3_ content. Herein, the NIR emission first increases up to the 1Nd glass with 1 mol % Nd_2_O_3_ but decreases thereafter for higher Nd_2_O_3_ concentrations in the 2–4Nd glasses. This can be appreciated by the plot shown in the inset of Figure 9 where the peak Nd^3+^ PL intensity at 1056 nm for the ^4^*F*_3/2_ → ^4^*I*_11/2_ transition of interest to laser applications is graphed vs the Nd_2_O_3_ concentration in the glasses. The steady decrease after 1 mol % Nd_2_O_3_ exhibits a linear trend as indicated by the regression analysis performed on the data points for the 1–4Nd glasses yielding a correlation coefficient *r* = −0.994. The data thus points to strong PL quenching at high Nd^3+^ concentrations translating into short Nd^3+^–Nd^3+^ interionic distances (Table 2). Ismail et al.^19^ conversely reported for multicomponent phosphate glasses with 60P_2_O_5_-8Al_2_O_3_-2Na_2_O-17K_2_O-(13 – *x*)BaO-*x*Nd_2_O_3_ with *x* = 0, 0.5, 0.75, 1.0, and 1.5 compositions that the PL always increased and thus ascribed it to negligible clustering. However, the current results resemble the reported by Sontakke et al.^4^ for glasses with (100 – *x*)(20.95BaO-11.72Al_2_O_3_-56.12P_2_O_5_-6.79SiO_2_-3.91B_2_O_3_-0.51Nb_2_O_5_) + *x*Nd_2_O_3_ compositions where the maximum PL was observed for 1 mol % Nd_2_O_3_ and decreased thereafter. Similar outcomes of PL quenching have been also observed for different types of laser phosphate glasses at relatively high Nd^3+^ concentrations in connection with energy transfer between Nd^3+^ ions (i.e., concentration quenching).^3,9,41^ The typical processes considered at the origin of the concentration quenching effect are the ^4^*F*_3/2_:^4^*I*_9/2_ → ^4^*I*_15/2_:^4^*I*_15/2_ donor–acceptor cross relaxation pathway and the ^4^*F*_3/2_:^4^*I*_9/2_ → ^4^*I*_9/2_: ^4^*F*_3/2_ donor–donor excitation migration (also called “hopping”) mechanism^3,4^ which are illustrated in Figure 10. As in prior studies by different researchers,^3,4,9,41^ additional information on ion–ion interactions is herein obtained from emission decay kinetics as considered next.

**Figure 11:**
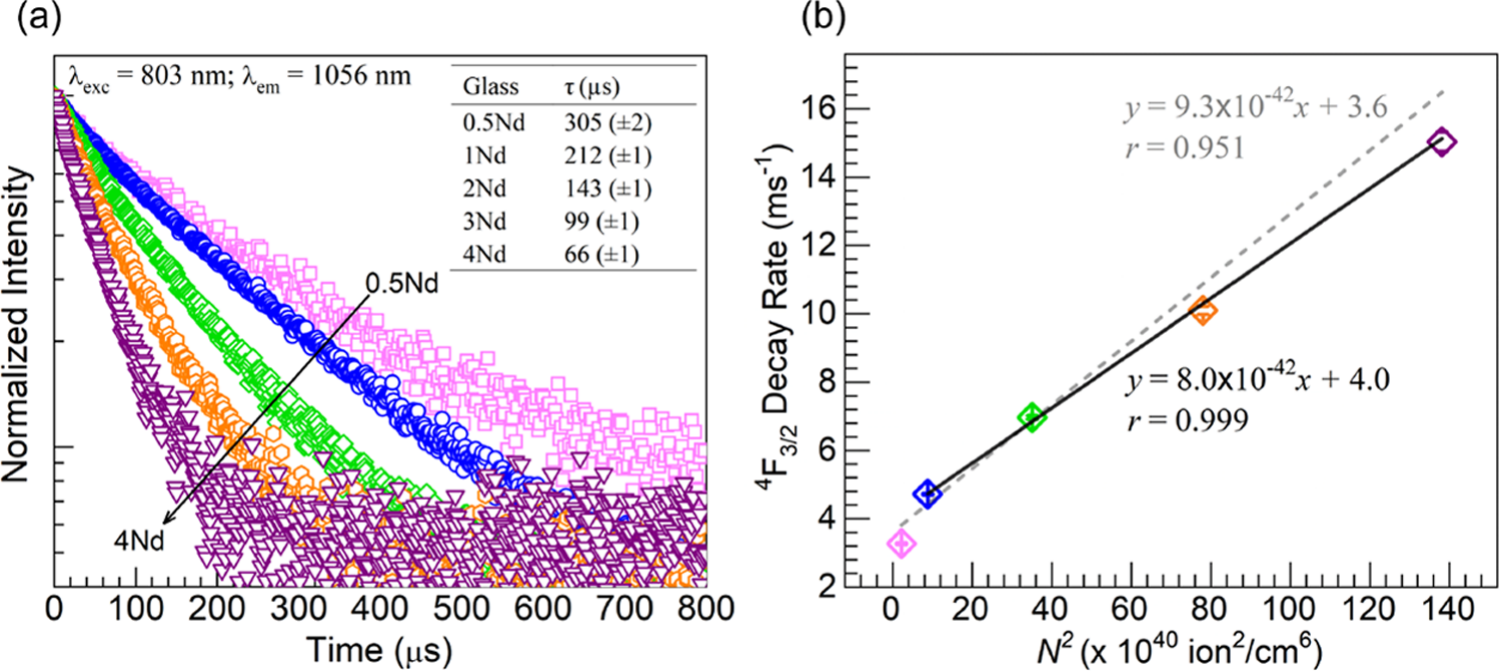
(a) Semilog plots of emission decay curves obtained for the 0.5Nd (squares-pink), 1Nd (circles-blue), 2Nd (diamonds-green), 3Nd (hexagons-orange), and 4Nd (triangles-purple) glasses under excitation at 803 nm by monitoring emission at 1056 nm. The table embedded as inset presents the ^4^*F*_3/2_ lifetimes (τ) estimated for Nd^3+^ ions in the glasses from exponential fits to the data. (b) Plot of Nd^3+4^*F*_3/2_ emission decay rates as a function of the square of the concentration (*N*^2^) of Nd^3+^ ions (*N* values listed in Table 2) in the glasses; the symbols are color-coded as in panel (a). The dashed and solid lines are linear fits to the data encompassing the 0.5–4Nd and 1–4Nd glasses, respectively (equations and correlation coefficients, *r*, displayed).

Figure 11a shows the Nd^3+^ emission decay curves obtained for the 0.5–4Nd glasses monitoring the ^4^*F*_3/2_ → ^4^*I*_11/2_ lasing emission at 1056 nm under excitation at 803 nm. The normalized data clearly shows that faster decays ensue with the increase in Nd^3+^ concentration in the glasses. The curves appear with exponential decay behavior and were thus fit following first-order decay kinetics^14^ with the function
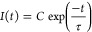
9where *I(t)* is the time-dependent emission intensity, *C* is a pre-exponential weight factor, and τ is the decay time. The corresponding Nd^3+4^*F*_3/2_ lifetimes deduced form the fits were 305 (±2), 212 (±1), 143 (±1), 99 (±1), and 66 (±1) μs for the 0.5Nd, 1Nd, 2Nd, 3Nd, and 4Nd glasses, respectively [also displayed in the table embedded in Figure 11a]. The obtained values are of the order of the determined experimentally for different Nd-containing phosphate glasses.^2,4,9,10,14,41^ Clearly, the decay times herein decreased continuously through the entire glass set. Thus, even though the PL intensity was highest for 1Nd, the glass still exhibited a shorter lifetime than the 0.5Nd with weaker emission (Figure 9). Interestingly, Sontakke et al.^4^ likewise reported the ^4^*F*_3/2_ lifetimes to decrease continuously in the entire concentration range considered for the (100 – *x*)(20.95BaO-11.72Al_2_O_3_-56.12P_2_O_5_-6.79SiO_2_-3.91B_2_O_3_-0.51Nb_2_O_5_) + *x*Nd_2_O_3_ glasses with *x* = 0.1, 0.3, 0.5, 1.0, 1.5, 3.0, 5.0 even though the PL intensity increased up to *x* = 1.0 and then decreased. Ramprasad et al.^9^ and Neelima et al.^41^ observed similar tendencies in laser phosphate glasses wherein the Nd^3+^ lifetimes were observed to decrease in connection with PL quenching. An approach to analyze the shortening of Nd^3+^ lifetimes in the context of concentration quenching in near-metaphosphate laser glasses is based on the empirical formula^3,4^
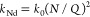
10where *k*_0_ is the zero-concentration decay rate, *k*_Nd_ the decay rate due to the increasing concentration of Nd^3+^ ions, *N* is the Nd^3+^ concentration, and *Q* is an empirically determined quantity for the glass, representing the concentration needed to decrease the lifetime to half of its zero-concentration limit. A linear relationship in this framework is then considered indicative of the prevalence of nonradiative relaxation of Nd^3+^ ions proceeding via the donor–donor excitation migration pathway (illustrated in Figure 10).^3,4^ Thus, Figure 11b shows a plot of the estimated Nd^3+4^*F*_3/2_ emission decay rates, *k*_Nd_ = τ ^–1^ [τ values shown in the Table embedded in Figure 11a], as a function of the square of the concentration (*N*^2^) of Nd^3+^ ions (*N* values listed in Table 2) in the glasses. In a first attempt, the data for all five Nd-containing glasses was fit by linear regression analysis which yielded a correlation coefficient *r* = 0.951 pointing to a weak correlation for the entire concentration range. However, considering that the PL intensity increased up to the 1Nd glass and decreased afterward (Figure 9), a second attempt was made to fit the data for the four 1–4Nd glasses as shown in Figure 11b. Therein, the value of *r* = 0.999 was obtained indicating a strong quadratic dependence for this Nd^3+^ concentration range, wherein the estimated Nd^3+^–Nd^3+^ mean distances decreased within the 15.0–9.48 Å range (Table 2). Accordingly, it is suggested that the concentration quenching observed in this regime proceeds primarily via the excitation migration or “hopping” mechanism. On the other hand, it seems that the lifetime decrease observed for the 1Nd glass relative to the 0.5Nd but that was accompanied by PL increase may reflect the contribution of donor–acceptor cross relaxation with lesser impact on emission intensity.

**Figure 12:**
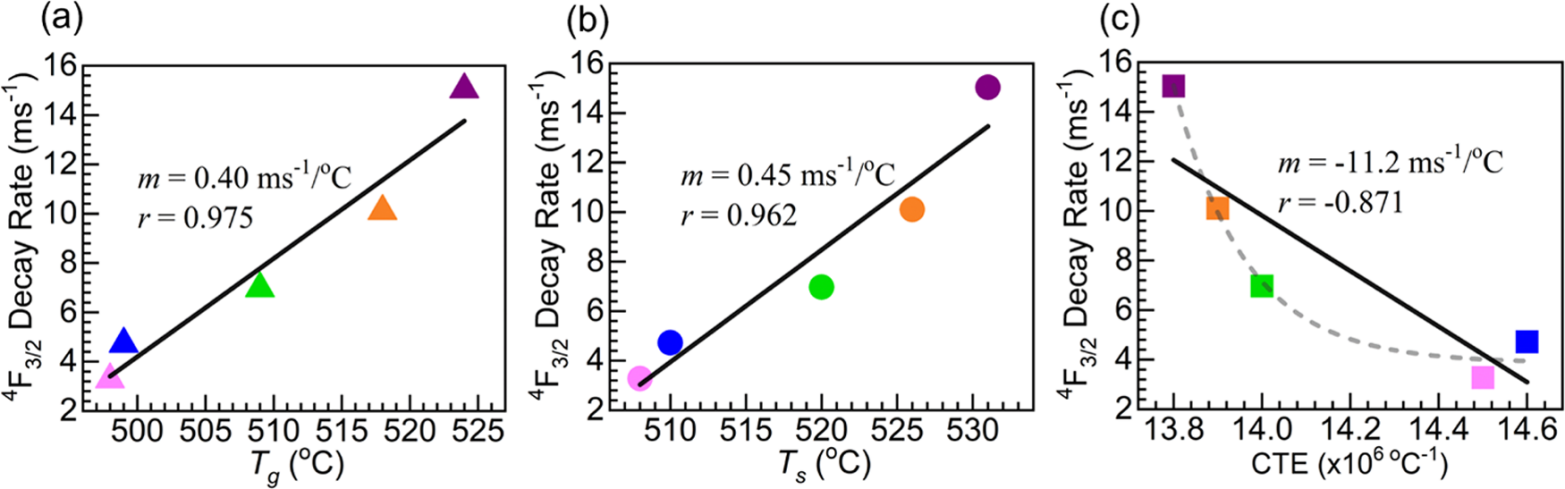
Plots of Nd^3+4^*F*_3/2_ decay rates as a function of (a) *T*_g_ (DSC), (b) *T*_s_ (dilatometry), and (c) CTE (dilatometry). The solid lines are linear fits to the data; slopes (*m*) and correlation coefficients (*r*) displayed. The dashed trace in panel (c) is a guide for the eye. The colors of the symbols in all panels represent each glass: 0.5Nd-pink, 1Nd-blue, 2Nd-green, 3Nd-orange, and 4Nd-purple.

At this point, we consider the possible connection between the different structural, thermal, and optical properties studied. The Raman spectroscopy and XPS assessment supported an increased depolymerization of the X-ray amorphous network with the increase in Nd_2_O_3_ content. Thereafter, the various thermal parameters scrutinized also showed reasonable agreement with a Nd_2_O_3_ concentration dependence. Then, while the UV–vis–NIR absorption increased steadily with Nd_2_O_3_ concentration, the NIR emission intensity was strongest for 1Nd_2_O_3_ mol % but decreased afterward. Nonetheless, the Nd^3+4^*F*_3/2_ decay rates always increased. Hence, an attempt is made to screen for a potential use of the latter intensive property, being independent of sample size and dimensions, with the *T*_g_, *T*_s_, and CTE as thermal parameters of interest reflecting the structural evolution linked to Nd^3+^ field strength effects. The resulting plots are shown in Figure 12a–c where the entire Nd-containing glass set is considered for completeness. It is interesting to note that reasonable linear correlation is observed between the Nd^3+4^*F*_3/2_ decay rates and the *T*_g_, followed by *T*_s_. The slopes for the plots with *T*_g_ and *T*_s_ in Figure 12a,b in fact are comparable. A large scattering of the experimental data points is however seen with the CTE in Figure 12c. Even though the CTE plot shows lack of linear correlation, this analysis suggests an interconnection between the environment of Nd^3+^ ions and glass thermal properties which are ultimately linked to the structural evolution of the glasses with increasing Nd_2_O_3_ content. Herein, the more straightforward *T*_g_ measurements by DSC showing best correlation may then offer an alternative to anticipate PL performance which may assist in the design of the materials for optical applications.

## 4 Conclusions

Recapitulating, the melting technique was employed to synthesize phosphate glasses containing NIR-emitting Nd^3+^ ions of interest to lasers and solar spectral converters to pursue a composition–structure–property study through comprehensive characterizations. The glasses were prepared having near-metaphosphate 50P_2_O_5_-(50 – *x*)BaO-*x*Nd_2_O_3_ nominal compositions with *x* = 0.5, 1.0, 2.0, 3.0, and 4.0 mol %, and studied thoroughly by density, XRD, Raman spectroscopy, O 1s XPS, DSC, dilatometry, optical absorption, and PL spectroscopy with emission decay rates assessment. The densities and molar volumes of the Nd-containing glasses exhibited increasing trends with Nd_2_O_3_ content. The concentration of Nd^3+^ ions consequently augmented in the 1.49–11.75 × 10^20^ ions/cm^3^ range leading to Nd^3+^–Nd^3+^ interionic distances shortening within the 18.9–9.48 Å range. The XRD evaluation supported the amorphous nature of the glasses, whereas Raman spectroscopy indicated that glass depolymerization was promoted by increasing Nd^3+^ ions concentration. Further, O 1s and P 2p XPS analysis supported the structural examination by Raman spectroscopy demonstrating the effects of an increase in NBOs following the replacement of 4 mol % BaO with an equal amount of Nd_2_O_3_.

The thermal evaluation by DSC then showed that the glass transition temperatures increased with Nd_2_O_3_ content in the glasses. Herein, a glass strengthening effect linked to the higher ionic field strength of Nd^3+^ ions compared to Ba^2+^ is considered underlying the thermal behavior. Further observed was a shift to higher temperatures for the onset of crystallization and a decreased susceptibility for crystallization with Nd_2_O_3_ content manifested as suppressed exotherms with decreasing magnitudes of the specific enthalpy change. Dilatometry also showed increasing softening temperatures in accord with the DSC evaluation supporting a stronger network was achieved with increasing Nd^3+^ concentration. Moreover, a trend of decrease in the coefficient of thermal expansion for the Nd^3+^-containing glasses was found indicating increased glass rigidities were realized despite depolymerization being induced. This trend also harmonizes with the increasing concentration of high-field strength Nd^3+^ ions leading to a tighter network while replacing Ba^2+^ ions.

The optical UV–vis–NIR absorption assessment showed a linear increase of Nd^3+^ absorption with neodymium content, thus supporting the effective incorporation of Nd^3+^ ions up to 11.75 × 10^20^ ions/cm^3^ corresponding to 4 mol % Nd_2_O_3_. The optical UV–vis assessment of glass absorption edges in the context of Tauc plots indicated overall higher band gap energies for the Nd-containing glasses relative to the undoped host, likely related to the higher field strength of Nd^3+^ ions compared to Ba^2+^. Further evaluation of the Urbach energies showed a decrement at low Nd_2_O_3_ content of 0.5 mol %, but consistently higher values for 1–4 mol % Nd_2_O_3_ linked with a tendency for increased structural disorder. Further on, the NIR emission intensity was highest for 1.0 mol % Nd_2_O_3_ and decreased thereafter. Further, the decay kinetics of the ^4^*F*_3/2_ emitting state Nd^3+^ ions analyzed revealed decreasing lifetimes for all Nd-containing glasses. The decay rate analysis with the square of the Nd^3+^ ion concentration pointed to the prevalence of excitation migration or “hopping” mechanism as central energy transfer pathway leading to PL quenching at high Nd^3+^ concentrations. Thus, even though the glass matrix effectively incorporates Nd^3+^ ions while becoming depolymerized, there is significant propensity for ion–ion interactions at high Nd^3+^ concentrations which are undesirable for optical applications requiring optimum emission output. In this sense, given the apparent interconnectedness and Nd_2_O_3_ concentration dependence, the measurement of the glass *T*_g_ by DSC may assist in projecting the Nd^3+^ decay rates impacting the PL performance. From the applications standpoint, given the effect of Nd^3+^ ions of supporting a stronger glass structure with enhanced thermal properties, a scientist desiring to optimize multiple parameters may then choose to balance optical performance with other properties such as glass stability or thermomechanical attributes.

## Data Availability

The data underlying this study are available in the published article.
